# Social-Emotional and Behavioural Difficulties in Children with Neurodevelopmental Disorders: Emotion Perception in Daily Life and in a Formal Assessment Context

**DOI:** 10.1007/s10803-022-05768-9

**Published:** 2022-10-03

**Authors:** Joanna Löytömäki, Marja-Leena Laakso, Kerttu Huttunen

**Affiliations:** 1 Faculty of Humanities/Research Unit of Logopedics, University of Oulu, P.O. Box 1000, 90014 Oulun yliopisto, Finland; 2 Department of Education, University of Jyvaskyla, Finland PL 35, 40014 Jyvaskylan yliopisto, Finland; 3 Department of Otorhinolaryngology, Head and Neck Surgery, Oulu University Hospital, Oulu, Finland; 4 Medical Research Center Oulu, Oulu, Finland

**Keywords:** Social development, Emotion recognition, Social-emotional difficulties, Behavioural problems, Parent, Professional

## Abstract

Children with neurodevelopmental disorders often have social-emotional and behavioural difficulties. The present study explored these difficulties in children (*n* = 50, aged 6–10 years) with autism spectrum disorder, attention-deficit/hyperactivity disorder and developmental language disorder. Parents, teachers and therapists evaluated children’s social-emotional and behavioural difficulties through a self-devised questionnaire and the Strengths and Difficulties Questionnaire. Additionally, the children, along with their typically developing age peers (*n* = 106), completed six emotion discrimination tasks. Analysis revealed some impaired emotion discrimination skills that were predictive for behavioural challenges in daily life and associated with the parent-reported existence of friends. Timely intervention in these children is needed, and it should also include emotion perception training.

**Supplementary Information:**

The online version contains supplementary material available at 10.1007/s10803-022-05768-9.

## Introduction

Social-emotional skills constitute a complex set of skills such as social awareness, relationship skills, responsible decision-making, empathy, prosocial understanding and emotion recognition and discrimination (Weissberg et al., 2015; Widen, 2016). Emotion recognition and discrimination skills are related both to social cognition abilities, such as theory of mind (ToM), and to linguistic skills. ToM is described as the ability to understand what others feel, know and think (Baron-Cohen et al., 2009). Sufficient ToM skills are important factors not only for the development of emotion recognition skills (e.g. Baron-Cohen et al., 2009; Loukusa et al., 2014) but also for peer acceptance. ToM skills have also been associated with less frequent aggressive and withdrawn behaviour (Caputi et al., 2012; Slaughter et al., 2002). Additionally, adept linguistic skills, including good pragmatic language abilities, are crucial for social-emotional functioning (Rautakoski et al., 2021) and especially for forming and maintaining peer relationships (Leonard et al., 2011). In sum, the overall ability of children to communicate and relate to other people determines how well they can form and maintain peer relationships, and this further predicts their educational attainment and quality of life (Denham, 2006).

The present study focuses on exploring the similarities in the emotion recognition and discrimination skills of children with autism spectrum disorders (ASD), attention-deficit/hyperactivity disorder (ADHD) and developmental language disorder (DLD). Another focus is to determine if possible delays in the development of emotion discrimination skills are reflected in behavioural problems that emerge in daily life. In the present article, the term ‘recognition’ is used to refer to the perception of emotions when no alternatives are available, whereas the term ‘discrimination’ is used to refer to the perception of emotions in forced-choice tests. ‘Perception’ is used to cover them both.

### Social-Emotional Difficulties in Children with Neurodevelopmental Disorders

Children with ASD, ADHD and DLD often experience similar difficulties in social-emotional development and particularly in emotion recognition (Taylor et al., 2015; Waddington et al., 2018). These disorders comprise closely interconnected genetic or hereditary factors (Bishop et al., 2017; Rommelse et al., 2010; Smoller et al., 2013; Weismer, 2013) and quite often co-exist with each other (e.g. Leitner, 2014; Loucas et al., 2008). These similarities have also affected diagnostic criteria, leading to changes being introduced in both the ICD-11 (WHO, 2019) and DSM-5 criteria (APA, 2013).

Difficulties in emotion recognition and behavioural problems often co-occur, and poor language skills are intertwined with them (Cohen & Mendez, 2009; Geurts & Embrechts, 2008; Mackie & Law, 2010; Shablack & Lindquist, 2019). Some of the typical emotion recognition difficulties demonstrated by children with neurodevelopmental disorders include, but are not limited to, poor recognition of facial expressions or emotions in the voice (Boucher et al., 2000; Griffiths et al., 2020; Kuusikko et al., 2009). Emotion recognition difficulties are well researched in ASD, and there is increasing evidence for them in children with DLD (Taylor et al., 2015). With regard to ADHD, some children have difficulties in emotion recognition (Demopoulos et al., 2013; Jusyte et al., 2017; Sjöwall, et al., 2013), although contrary findings also exist (see e.g. Borhani & Nejati, 2018). Additionally, Demopoulos et al. (2013) compared the emotion recognition skills of children with ADHD and ASD and found that both groups had significantly poorer skills than typically developing (TD) peers in recognising emotions from face and voice. Diagnoses of ASD, ADHD and DLD commonly overlap, and children diagnosed with any of these are frequently referred to speech therapy. There have been studies of each diagnostic group of children with emotion recognition problems. Of these studies, some have focused only on one diagnostic group (Griffiths et al., 2020; Staff et al., 2022) and some have compared two of them (Taylor et al., 2015); nevertheless, even though these diagnoses commonly co-occur, none have yet included all three groups.

Behavioural problems usually accompany social-emotional difficulties emerging in both family and early education contexts (Kurki et al., 2017; Morris et al., 2007). They are typically divided into *internalising* symptoms, which include problems with emotional life, such as depression or anxiety, and *externalising* symptoms, such as behavioural or hyperactivity problems (Helland & Helland, 2017; Yew & O’Kearney, 2013). Further, an inability to ask for help or unwillingness to help others may be signals of poor prosocial skills (Deschamps et al., 2014). Charman et al. (2015) found that both children with ASD and those with language impairment exhibit similar levels of behavioural problems, based on an assessment by teachers using the Strengths and Difficulties Questionnaire (SDQ). Children with ADHD have also been demonstrated to show behavioural symptoms, and according to some studies, co-existing ASD may influence the severity of behavioural functioning of children with ADHD (e.g. Thomas et al., 2018).

Research has suggested that children with ASD, ADHD and DLD demonstrate poor peer relationship skills and even experience loneliness, which possibly stems from their multiple social-emotional difficulties (Forrest et al., 2018; Hoza, 2007; Petrina et al., 2014; Smit et al., 2020). Overall, children’s emotion knowledge is associated with peer acceptance and success in school (Voltmer & Von Salisch, 2017). Without appropriate intervention, problems in peer relationships can persist into adolescence and even adulthood, for example, in individuals with DLD (Conti-Ramsden et al., 2013).

Ever more data is accumulating on the social-emotional and behavioural difficulties exhibited by children with neurodevelopmental disorders and on how these difficulties are intertwined. In the research field, practically no studies on the occurrence of social-emotional difficulties in the daily lives of children have simultaneously covered diagnoses of ASD, ADHD and DLD.

### Aims and Hypotheses

The present study aimed to obtain multifaceted information about the kinds of social-emotional and behavioural difficulties children with ASD, ADHD and DLD exhibit in their daily lives and explore whether they share similar challenges.

The first research question sought to identify the existence and severity of emotion recognition difficulties in children with neurodevelopmental disorders in their daily lives as reported by parents, teachers and therapists, and if these difficulties differed by diagnosis. Typical emotion recognition difficulties often demonstrated by children with neurodevelopmental disorders include, for example, poor recognition of facial expressions (Griffiths et al., 2020; Kuusikko et al., 2009; Spackman et al., 2005; Staff et al., 2022; Taylor et al., 2015) or emotions in the voice (Boucher et al., 2000; Corbett & Glitten, 2000).

#### Hypothesis 1

Regardless of their diagnosis, children with neurodevelopmental disorders have emotion recognition difficulties in daily life.

The second research question aimed to explore the type of social-emotional and behavioural difficulties exhibited by children with neurodevelopmental disorders in their daily lives, as reported by their parents, teachers and therapists. To answer this question, the adults were asked to report on situations in which these children’s social-emotional difficulties emerge and the consequences of these situations. This was built on earlier research that has shown that children with neurodevelopmental disorders demonstrate wide range of social-emotional challenges in their daily lives, as well as problems with ToM (Bakopoulou & Dockrell, 2016; Baron-Cohen et al., 2009; Loukusa et al., 2014). Furthermore, behavioural difficulties have been demonstrated to stem from multiple social-emotional difficulties (Charman et al., 2015; Kurki et al., 2017; Morris et al., 2007; Thomas et al., 2018; Yew & O'Kearney, 2013).

#### Hypothesis 2

Children with neurodevelopmental disorders exhibit a wide variety of social-emotional and behavioural difficulties in their daily lives, as perceived by parents, teachers and therapists.

The third aim of this study was to assess the performance level of children with neurodevelopmental disorders in emotion discrimination tests and tasks and compare it with that of their TD age peers. There is evidence of difficulties in emotion discrimination skills in children with ASD and DLD (Taylor et al., 2015; Waddington et al., 2018) and in some children with ADHD (Demopoulos et al., 2013). These findings imply that the difficulties in emotion discrimination skills associated with the three diagnoses in focus in the present paper may be similar to each other.

#### Hypothesis 3

In comparison with their TD and age matched peers, children with neurodevelopmental disorders show delayed emotion discrimination skills.

The fourth aim of the present study was to examine whether possible delays in emotion discrimination skills together with expressive language delays explain children’s behavioural problems and lack of friends. Problems in language and emotion recognition skills are commonly demonstrated in children with neurodevelopmental disorders and these problems are further associated with behavioural difficulties (Cohen & Mendez, 2009; Cooper et al., 2020; Geurts & Embrechts, 2008; Mackie & Law, 2010; Shablack & Lindquist, 2019). Furthermore, poor peer relationship skills and even loneliness possibly stem from emotional difficulties and are prevalent in children with neurodevelopmental disorders (Forrest et al., 2018; Hoza, 2007; Petrina et al., 2014; Smit et al., 2020). In a fairly recent review, loneliness in particular was found to have negative consequences on the mental health and overall development of children with neurodevelopmental disorders (Kwan et al., 2020).

#### Hypothesis 4

Together with the delay of expressive language skills, a delay in emotion discrimination skills explain behavioural problems and lack of friends in children with neurodevelopmental disorders.

## Methods

The study protocol was approved by the Ethical Committee of the Northern Ostrobothnia Hospital District, Finland, and it conforms with the Declaration of Helsinki as revised in 2000 (World Medical Association, 2001). The assent of the children and informed written consent from their parents, was obtained before their participation in the study. Additionally, written consent was obtained from those children who were able to read the research notice, which had been specifically adjusted to their language level and provide consent themselves.

The following inclusion criteria were used for recruiting the participants: (1) diagnosis of ASD, ADHD or DLD set in the tertiary health-care setting; (2) difficulties in recognising emotions from the face, voice or both, as reported by parents or speech and language therapists, occupational therapists, psychologists, early education teachers or school teachers; (3) age between 6 and 10 years; (4) performance IQ over 85; (5) monolingual Finnish-speaking family; and (6) sufficient vision, hearing, motor and attention skills to complete the tests and tasks presented to the children. The information needed to fulfil these inclusion criteria was gathered from parents.

They were also asked to provide information about their respective child’s diagnoses and medication, and, if needed, they were asked to seek further information from the hospital responsible for the care of their child. Children with psychiatric diagnoses such as depression were not included.

### Participants

A total of 50 children diagnosed with ASD, ADHD or DLD formed the clinical group of this study (Table 1). Almost all the participants lived in urban areas in different geographical regions of Finland. They were recruited through hospitals, privately practising speech and language and occupational therapists, psychologists, schools and parent organisations by disseminating information about the study and its inclusion criteria. Additionally, a group of TD 6- to 10-year-old children (*n* = 106, 20 to 22 children per age group), was recruited from day-care centres and schools to serve as a neurotypical reference group for the self-constructed emotion discrimination tasks, for which no published age norms were yet available. The mean age of both the clinical group and the comparison group of TD age peers was 8.02 years. Neither age nor the male : female ratio significantly differed by diagnosis. However, in the clinical group, the number of males was significantly higher than that in the TD age peer group (Fisher’s exact test, *p* < 0.001).

**Table 1:** Characteristics of the Children with Neurodevelopmental Disorders and Typical Development

	ASD (*n* = 20)	ADHD (*n* = 17)	DLD (*n* = 13)	Total (*n* = 50)	TD (*n* = 106)
Males:females (*n*)	18:2	14:3	9:4	41:9	47:59
Age, mean, (*SD*)	8.25 (1.21)	8.06 (1.30)	7.62 (1.61)	8.02 (1.25)	8.02 (1.42)
Single diagnosis (*n*)	11	9	10	30	N/A
Comorbid diagnoses (*n*)	9	8	3	20	N/A

*ASD—*Autism spectrum disorder, *ADHD*—Attention-deficit/hyperactivity disorder (including three children with *ADD*, Attention-deficit disorder), *DLD*—Developmental language disorder, *TD*—Typically developing, *N/A*—Not applicable

In the clinical group, 13 children had been diagnosed with specific language impairment according to ICD-10 criteria (National Institute for Health and Welfare, 2011), which are currently used in clinical practice in Finland. However, the contemporary label of DLD is used in this article, since it does not exclude either cognitive or executive function, or motor, social or emotional difficulties, which may co-occur with language impairment. The term DLD is included in DSM 5 (APA, 2013) and will also be increasingly used clinically and in research settings when the ICD-11 (WHO, 2019) classification is adopted in different countries.

Of the 50 participants who had been diagnosed by a phoniatrician or child psychiatrist, 30 children had a single diagnosis (ASD, ADHD or DLD) and 20 had at least one comorbid diagnosis in addition to the diagnosis that was judged in this study to be the primary one. In the case of comorbid diagnoses, the primary diagnosis was determined by the first and last authors of this study, and the decision was based on the child’s symptom profile from information derived from medical records provided by their parents. If no additional information was available, the decision was based on the scientific and clinical literature. For example, if a child had been diagnosed with both ADHD and ASD according to standard clinical practice in Finland (Sumia et al., 2016), ASD was determined to be the primary diagnosis. If a child was under medication for ADHD and additionally had DLD, ADHD was considered the more severe condition of the two and was therefore classified as the primary diagnosis. Seven of the participants also had subsidiary diagnoses, such as a motor function disorder (e.g. F82 in ICD-10) or Tourette’s syndrome. These additional disorders did not affect the cognitive performance of the children, and such participants were therefore included in this study.

Of the 20 children with comorbid diagnoses, six had been diagnosed with both ASD and ADHD, seven had ADHD and DLD, three had ASD and DLD, and four had all three diagnoses. As many as 40% of the participants, therefore, had multiple diagnoses, although, for example, ASD would preclude a diagnosis of ADHD, and both ADHD and DLD would preclude ASD conditions, according to ICD-10. Most (17) of the 20 children with ASD had been diagnosed with Asperger’s syndrome, and three had been diagnosed with pervasive developmental disorders unspecified. Three children had attention-deficit disorder (ADD); however, since ADD is categorised under the diagnostic code of ADHD, the term ADHD therefore also covered those diagnosed with ADD.

### Procedures

Data on the clinical group were gathered using questionnaires filled out by all the adults, and the children completed individual tests and tasks. Data on the TD group were gathered only by direct testing. Both quantitative and qualitative methods were used in the data analysis. All individual test sessions were both voice-recorded and video-recorded so that the scoring could be checked. All the tests, tasks and questionnaires used for collecting the data, and their psychometric properties, are summarised in Online Resource 1.

### Data Collection Through Questionnaires

Parents and speech and language therapists, occupational therapists or teachers assessed the ability of the children to recognise emotions from the face, voice and bodily postures (expressions) using a visual analogue scale (VAS). The range of the VAS was 0–100, with 0 meaning that the child had no ability at all to recognise emotions and 100 meaning that the child excelled in emotion recognition skills. The following questions were asked in the questionnaire: ‘At the moment, how difficult is it for your child to recognise emotions from facial expressions?’, ‘At the moment, how difficult is it for your child to recognise emotions from tone of voice (e.g. raising one’s voice, kind or irritated tone of voice)?’ and ‘At the moment, how difficult is it for your child to recognise emotions from bodily postures and gestures (e.g. retracting when feeling disgusted, shaking a fist and blowing kisses)?’. By using the VAS scale, it was possible to obtain information on how the adults perceived the children’s emotion recognition skills in real life social situations. Visual modality (conveying facial expressions) is the most commonly studied of these modalities when emotion recognition is explored. Inclusion of other modalities, such as tone of voice and bodily postures, are also just as important, as they too have been reported to be a challenge in neurodevelopmental disorders, and neural processes underlying them are different from those used in the recognition of facial expressions (De Gelder, 2009; Liebenthal et al., 2016).

The questionnaire also contained open-ended questions asking the adults to provide examples of situations in which they had observed emotion recognition difficulties in their child. They were then asked to describe how these problems affected their child’s daily life (see Online Resources 2 and 3). The parents also reported the number of friends who visited their child each week, and the number of friends the child visited every week.

Parent and teacher versions of the SDQ (Goodman, 1997) for assessing 4- to 17-year-olds were used to explore how the adults perceived the children’s behavioural problems and prosocial skills. The SDQ is a 25-item Likert-type scale questionnaire with five subscales: emotional symptoms, behavioural problems, hyperactivity, peer relationship problems and prosocial behaviour. Each subscale comprises five items scored from 0 to 2, with a higher score indicating greater problems. However, for the prosocial behaviour subscale, a higher score indicates better skills. The higher the total score of SDQ (0–40), excluding the prosocial scale, the more difficulties are experienced by a child.

SDQ has been validated in Finnish 4- to 9-year-olds (Borg et al., 2014) and 7- to 12-year-olds (Koskelainen et al., 2000), and it has been found to have acceptable reliability. The Finnish cut-off points [the 90th percentiles, as Goodman (1997) recommended], based on the reports of 2635 parents and 2242 teachers, provided by Borg et al. (2014), were used in this study.

### Assessment of Emotion Discrimination and Linguistic Skills

The skills of the children with neurodevelopmental disorders were individually assessed at their homes, day-care centres, schools or clinics, depending on where it was convenient for their families to arrange a time and place for the tests. The tasks were administered in an identical manner across the different test settings for the clinical group. To determine the age-appropriate performance level, the TD age peers also completed the same tasks, except for the Boston Naming Test. In the TD group, the 6- to 8-year-olds were tested individually, but the 9- to 10-year-olds were tested in groups in their school classrooms to save time. In the group tests, stimuli were presented using a computer, loudspeakers and a data projector, and the children noted their responses on paper. The results of all the emotion discrimination tasks for the 7- to 8-year-old TD children (*n* = 42) tested individually did not significantly differ from those for the 7- to 8-year-old TD children (*n* = 43) tested in groups (results previously reported elsewhere).

All the results for the clinical group are expressed as the delay compared with the results of the TD age peers; each child’s discrimination result was compared with the mean result of the TD controls of the same age (in years). The participants’ skills were assessed using forced-choice tasks for the discrimination of facial expressions and emotional tone of voice and one face-voice matching task. Although these results have already been reported in an earlier article (Löytömäki et al., 2020), they are included here to determine if the results of direct assessment of emotion discrimination skills conform to the behavioural problems that emerge in everyday contexts.

Two tasks were constructed for exploring the discrimination of the emotional tone of voice. In the emotional nonsense word task, the children listened via computer speakers to 18 items spoken by an actress and recorded in a professional recording studio. Three nonsense words that corresponded to three basic emotions (happy, sad and angry) were used as single words in half the stimuli, and in the other half, they were embedded in a semantically neutral carrier phrase (‘Now I say …’), with the prosody of the carrier phrase matching the tone of voice of the nonsense word. The second task consisted of 11 items for recognising the emotional tone of voice in sentences in which the semantic content aligned with the emotion. All six basic emotions (joy, anger, sadness, fear, surprise and disgust) as well as ashamed and neutral tone of voice were included in this task. In both tasks, the participants could answer verbally or point to emoji placed in front of them.

To assess the discrimination of facial expressions, two tasks were constructed: one comprising eight photographs in paper form or on a computer screen and the other one comprising eight video clips. Both tasks included the six basic emotions as well as ashamed and neutral facial expressions. Facial expressions for these tasks were recorded in a professional photographer’s studio by one professional actress, one actor and two children (aged 10 and 11 years). In both tasks, the test administrator orally provided the participants with four verbal labels as response alternatives. In addition to these two tasks, the Faces submodule of the Finnish version of the computerised Frankfurt Test and Training of Facial Affect Recognition 2 (FEFA 2; Bölte et al., 2013) test was also used. The Faces submodule consists of 50 photographs depicting seven different emotions (the six basic emotions and a neutral facial expression). In this test, seven written labels were provided for the participants to choose from (they were read out loud to those who could not yet read).

In the final task, participants were requested to match facial expressions with the emotional tone of voice for 11 items (which again included the six basic emotions and ashamed and neutral expressions), with happiness, anger and sadness all occurring twice. The items were presented using a computer and desktop loudspeakers. The children were asked to point at a photograph depicting a facial expression that matched the sentence they heard. The semantic content of the sentences was aligned with the emotions used in them.

The linguistic skills of the participants with neurodevelopmental disorders were explored in the expressive language domain using the validated Finnish version (Laine et al., 1997) of the Boston Naming Test (Kaplan et al., 1983). Neither semantic nor phonemic cueing was used, as vocabulary size, not naming ability, was the target for the test.

### Analyses

Answers to the two open-ended questions concerning the children’s emotion recognition difficulties in daily life and their consequences were analysed using qualitative content analysis. Expressions indicative of emotion recognition difficulties were considered as the units of analysis (see Kyngäs & Kaakinen, 2019). A data-driven categorisation of the responses into larger themes was performed first. The parents’ and the therapists and teachers’ answers were separately analysed. Any discrepancies in categorisation between the two researchers analysing the data (the first and last authors) were resolved by discussion.

SPSS versions 26 and 27 were used for all the quantitative analyses. Cronbach’s alpha and the KR-20 method (for raw data representing nominal scale) were used to explore the internal consistency of different variables. Pearson’s correlation coefficient was used to explore the inter-rater reliability of VAS ratings on emotion recognition and the SDQ total score, and Spearman’s rank correlation coefficient to determine the concordance between the adult groups SDQ subscale scores, as they represented an ordinal measurement scale. The assumptions of the two-way ANOVA were met and it was therefore used for comparing the adult-reported emotion recognition skills (VAS) and parent-reported SDQ results of the three diagnostic groups. To identify differences in emotion discrimination skills between children with different diagnoses, one- and two-way ANOVA were used. In the one-way ANOVA analyses, either Bonferroni or Dunnett’s T3 correction was used for controlling for family-wise errors when several groups were compared. However, when children with a neurodevelopmental diagnosis (the ‘clinical group’) were compared with their TD age peers, independent samples *t*-test was used. Cohen’s *d* and partial eta squared (η^2^) were used to express effect sizes that illustrated the magnitude of between-group differences. Linear regression analysis was used to determine the degree to which the possible delay in emotion discrimination skills would predict the SDQ total score. With the Mann–Whitney *U* test, the differences between existence of friends and delays in emotion discrimination skills were explored. Lastly, logistic regression analysis was performed to determine the risk of having no friends in association with the magnitude of delay in emotion discrimination skills.

## Results

The emotion recognition skills of children with neurodevelopmental disorders as assessed by adults with VAS, are presented first. Then, the adults’ perceptions of the children’s daily life social-emotional difficulties, and the results of SDQ, are presented. Next, the results of the emotion discrimination tasks are reported in terms of if there were delays in children with neurodevelopmental disorders in comparison with TD age peers and if the delay differed by diagnosis. Third, the results of delays in expressive language and emotion discrimination skills in predicting behavioural problems, are presented. Finally, the existence of friends of children with neurodevelopmental disorders, and if emotion discrimination delays predicted the lack of friends, is described.

In the clinical group, data from therapists or teachers could not be obtained for nine children, as these children were not currently undergoing therapy and/or they were not yet at school. As there were fewer speech and language therapists (*n* = 13) and occupational therapists (*n* = 8) than teachers (*n* = 23), and because for only four children more than one therapist or teacher completed the questionnaires, all scores were pooled and averaged. Therefore, these respondents are hereon referred to as one group, namely ‘professionals’ (*n* = 38–41).

### Emotion Recognition Skills Assessed by Parents and Professionals Using VAS

The internal consistency (Cronbach’s alpha) of children’s emotion recognition skills based on three VAS scales of the parental assessment (*n* = 49) was 0.779, and 0.908 for the professionals’ assessment (*n* = 38). The cross-informant agreement between parents and professionals was moderate, although it did not quite reach statistical significance with regard to assessing the children’s ability to recognise emotions from facial expressions (Pearson’s *r*(38) = 0.309, *p* = 0.052). Additionally, the agreement between parents and professionals for the assessment of emotion recognition from tone of voice or bodily postures was poor [*r*(36) = − 0.079, *p* = 0.639 and *r*(35) = − 0.201, *p* = 0.232, respectively].

**Fig. 1:**
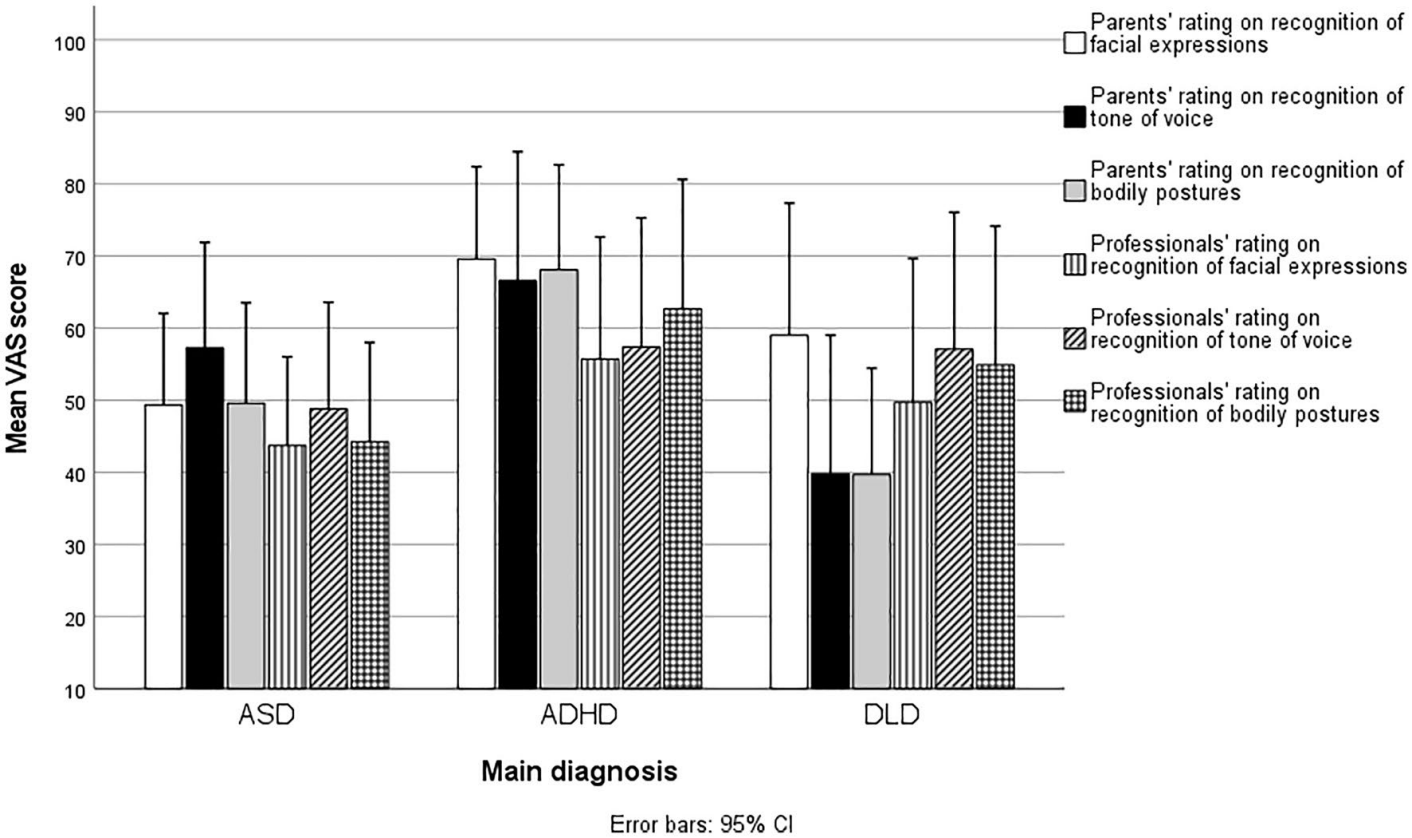
Parents’ and Professionals’ Mean VAS Rating of Emotion Recognition Skills of Children with Neurodevelopmental Disorders. Higher scores indicate better skills

The means and corresponding 95% *CI* values of the VAS scores assigned by parents (*n* = 49) and professionals (*n* = 41) for the children with neurodevelopmental disorders can be found in Fig. 1. The mean parental scores ranged from 53 to 58, and the mean professional scores ranged from 49 to 53. None of the parents’ or professionals’ three VAS scores indicated significant differences by diagnosis (*F*(2, 38) = 0.410–3.058, *p* = 0.057–0.667, η^2^ = 0.022–0.120).

### Parents’ and Professionals’ Reports on Children’s Social-Emotional Difficulties in Daily Life

After the kind of situations in which the emotion recognition difficulties in daily life occurred and the kinds of problems they posed had been analysed with content analysis, they were found to match the ToM classification of Baron-Cohen et al. (2009). Therefore, the data indicating issues related to ToM were further classified into cognitive and affective ToM.

With regard to the first question, both parents and professionals reported most problems to occur in situations in which affective ToM was required (Online Resource 2). These situations comprised a total of 59% of all the mentions of the parents and half of those of the professionals. The second largest theme representing both parents’ and professionals’ answers was cognitive ToM. After cognitive and affective ToM, the other two major themes identified in the responses to the first question were ‘social difficulties’ and ‘other problems’.

The second question concerned the problems these emotion recognition difficulties caused for the children in their daily lives (Online Resource 3). The responses to this question were also fairly similar between the parents and professionals. Almost half (40%) of the parents and 33% of the professionals reported emotion recognition problems to cause the children ‘social difficulties’. Other themes for the second question were ‘poor emotion regulation’ and ‘other problems’.

### Behavioural Problems Assessed with SDQ

The cross-informant Spearman rank correlation coefficient (ϱ) between the parent-reported SDQ total score and the professional-reported total score was low [*r*(39) = 0.231, *p* = 0.141]. When examining the five SDQ subscales, the correlation coefficients were as follows: 0.429 (*p* = 0.005) for the emotional symptoms subscale, 0.226 (*p* = 0.150) for the behavioural problems subscale, 0.362 (*p* = 0.019) for the hyperactivity subscale, 0.372 (*p* = 0.015) for the peer relationship problems subscale and 0.359 (*p* = 0.021) for the prosocial behaviour subscale.

**Fig. 2:**
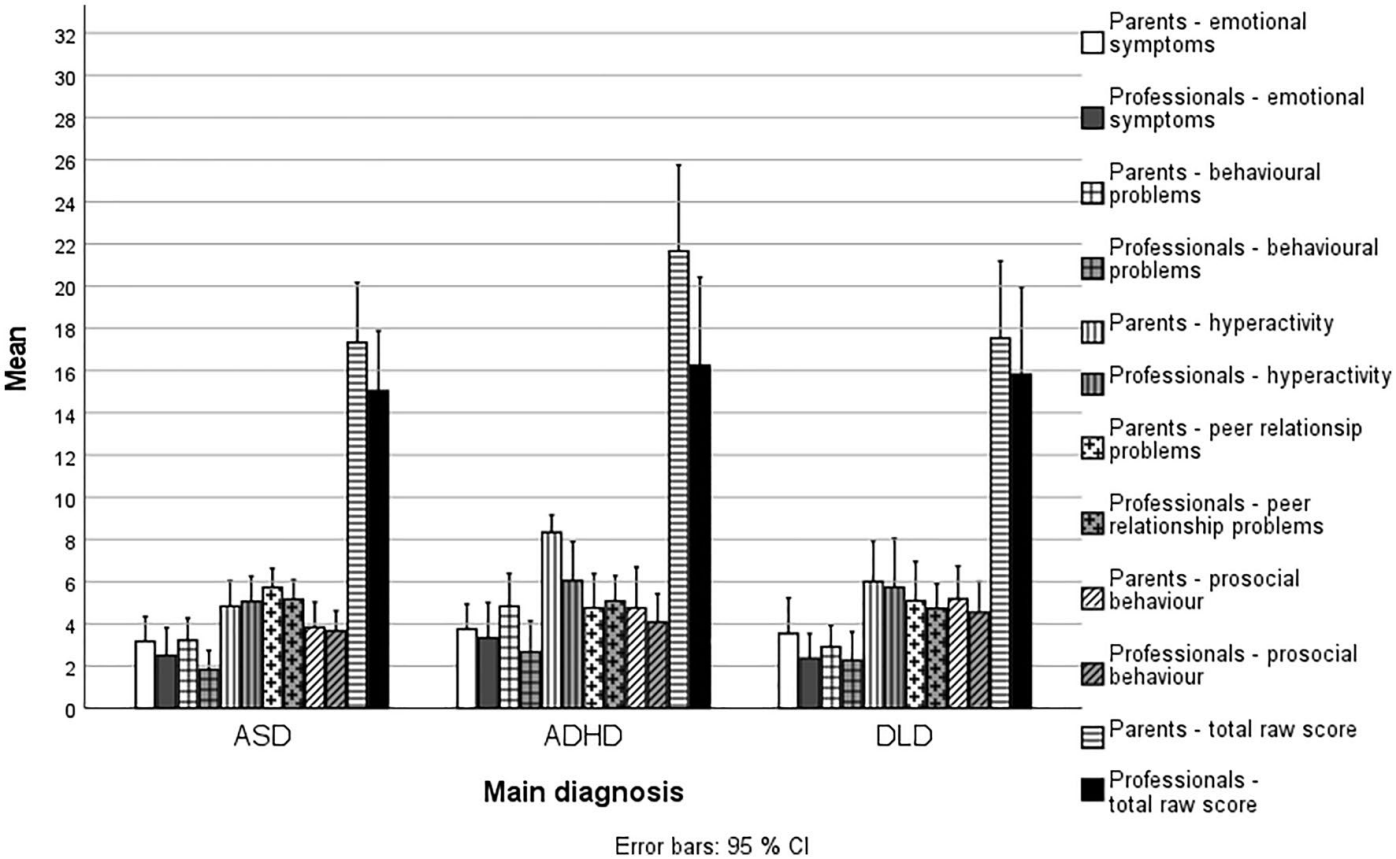
Mean (with *SD*) SDQ Subscale and Total Raw Scores of the Children with ASD, ADHD and DLD as Reported by the Parents (*n* = 49) and the Professionals (Child-by-Child Mean Across the Respondents, *n* = 41). SDQ = Strengths and Difficulties Questionnaire (R. Goodman, 1997). The total score of the SDQ can vary between 0–40, and the 90th percentile of both the parent- and teacher-reported norm scores for Finnish 4–9-year-old children is 12. Higher scores indicate bigger problems on all subscales except the prosocial subscale

All the SDQ scores assigned by the parents and professionals grouped by the children’s diagnoses are shown in Fig. 2 and Table 2. The SDQ scores show that the children in the clinical group had substantially more behavioural difficulties than the TD population of Finnish children.

**Table 2:** SDQ Total Scores of Children with ASD, ADHD and DLD as Categorised into ‘at or Above the 90th Percentile’ and ‘Within Age Norms’

Parents as informants – SDQ total score	ASD (*n* = 20)	ADHD (*n* = 17)	DLD (*n* = 13)	Total (*n* = 50)
At or above the 90th percentile (*n*)	18 (90%)	16 (94%)	11 (85%)	45 (90%)
Within age norms (*n*)	2 (10%)	1 (6%)	2 (15%)	5 (10%)

Professionals as informants – SDQ total score	ASD (*n* = 18)	ADHD (*n* = 12)	DLD (*n* = 11)	Total (*n* = 41)
At or above the 90th percentile (*n*)	13 (72%)	10 (83%)	8 (73%)	31 (76%)
Within age norms (*n*)	5 (28%)	2 (17%)	3 (27%)	10 (24%)

*SDQ* = Strengths and Difficulties Questionnaire (R. Goodman, 1997). Total scores are categorised according to the 90th. percentile reported in the Finnish validation study of Borg et al. (2014). The total score of the SDQ can vary between 0–40, and the 90th. percentile of both the parent- and teacher-reported norm scores for Finnish 4–9-year-old children is 12

The children in the clinical group were compared with each other based on their diagnosis. According to the parental SDQ reports, the mean total score of the children with ASD was 18 (*SD* = 5.59). In children with ADHD, it was 21 (*SD* = 6.16), and in children with DLD, it was 16 (*SD* = 5.8). There were neither main effects of diagnosis, age or sex nor their interaction in total scores, as assessed by the parents (from *F*(2,35) = 0.045, *p* = 0.833, η^2^ = 0.027 to *F*(2,44) = 1.848, *p* = 0.101, η^2^ = 0.144) and the professionals (from *F*(2,26) = 0.016, *p* = 0.985, η^2^ = 0.0125 to (*F*(2,35) = 1.081, *p* = 0.407, η^2^ = 0.070). However, in the case of parent-reported hyperactivity subscale score, ANOVA showed a main effect of diagnosis (*F*(2, 44) = 5.037, *p* = 0.011, η^2^ = 0.186), with the children with ADHD having higher scores (more symptoms) than children with ASD (*p* < 0.001) and DLD (*p* = 0.009), but there were no interactions between age, sex or diagnosis.

### Emotion Discrimination Skills Assessed by Tests and Tasks

Two-way ANOVA showed that there was a main effect of age in all tasks (nonsense words *F*(4) = 6.339, *p* < 0.001, η^2^ = 0.149; meaningful sentences *F*(4) = 2.615, *p* = 0.038, η^2^ = 0.068; FEFA 2 test *F*(4) = 9.199, *p* < 0.001, η2 = 0.202; photographs *F*(4) = 7.682, *p* < 0.001, η2 = 0.176; and video clips *F*(4) = 3.818, *p* = 0.006, η2 = 0.096) apart from the matching task (*F*(4) = 0.892, *p* = 0.471, η2 = 0.024). A main effect of sex was also found in two tasks: the FEFA 2 test, *F*(1) = 4.754, *p* = 0.031, η2 = 0.032, with boys having the better result, and the matching task *F*(1) = 5.061, *p* = 0.026, η2 = 0.034, with girls having the better result. No Age x Sex interaction effects were found in any of the six variables indicating raw scores of emotion discrimination.

**Fig. 3:**
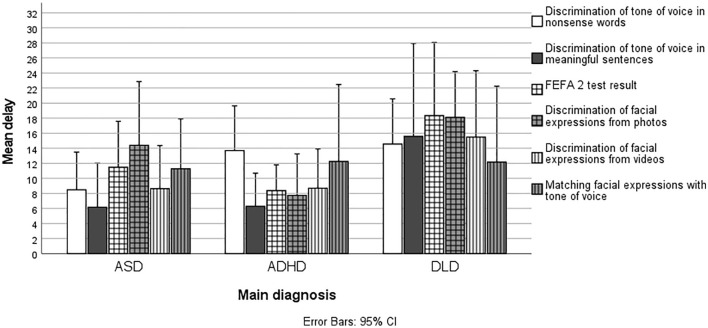
Emotion Discrimination Skills of the Children with ASD, ADHD and DLD Compared with their TD Age Peers (Mean Delay in Percentage Points). The results are expressed as mean delays compared to TD age peers, as the age of the participants in the clinical group varied from six to ten years

Independent *t*-tests showed that, compared with TD age peers, children in the clinical group had a slight but significant delay in all six emotion discrimination tasks (Fig. 3). The delay was 11 percentage points on average. The largest statistically significant delay compared with the TD children was found for the task in which facial expressions were discriminated from photographs (11 percentage points; *t*(152) = − 4.050, *p* < 0.001, *d* = 0.701), and the smallest delay was found for the task in which emotions were discriminated from meaningful sentences (5 percentage points; *t*(64.85) = − 2.052, *p* = 0.044, *d* = 0.423).

The delay in the emotion discrimination skills of children with only one diagnosis (*n* = 30) did not significantly differ from that of children with at least one comorbid diagnosis (*n* = 20) for any of the six emotion discrimination tests and tasks administered (from *t*(48) = − 1.249, *p* = 0.223 to *t*(47) = 0.112, *p* = 0.911).

### Associations Between Children’s Emotion Discrimination, Expressive Language and Behavioural Problems

Linear regression analysis was used to explore if the children’s emotion discrimination skills could predict their SDQ subscale scores. As the SDQ subscale scores assigned by the parents and the professionals correlated significantly in four out of five subscales, a linear regression analysis was performed only for the parental subscale scores. Additionally, the diagnosis was held constant in all the models because it had a stabilising effect. Sex was added into the models but was not a significant variable in any of them.

With the SDQ subscale scores as dependent variables, four models were found to be significant. The analysis revealed that when the delay variables of discriminating emotions from facial expressions in photographs and matching facial expressions with the emotional tone of voice were entered into the same model, they significantly predicted the children’s reported scores on two SDQ subscales: the behavioural problems subscale (*R*^*2*^ = 0.232, *p* = 0.011 for delay in discriminating emotions from photographs; *p* = 0.022 for delay in matching facial expressions with the emotional tone of voice) and the prosocial behaviour subscale (*R*^*2*^ = 0.255, *p* = 0.018 for delay in discriminating emotions from photographs; *p* = 0.004 for delay in matching facial expressions with the emotional tone of voice). In addition, the delay in discriminating emotions from video clips was found to be a significant predictor for the reported scores on the emotional symptoms subscale (*R*^*2*^ = 0.180, *p* = 0.009). Further, the delay of matching facial expressions with the emotional tone of voice significantly predicted the children’s reported scores in the hyperactivity subscale (*R*^*2*^ = 0.376, *p* < 0.001). All these findings indicate that the better the emotion discrimination ability, the fewer the behavioural difficulties (or the better the prosocial behaviour) that were reported. However, no significant predictors were found for the peer relationship problems subscale. A delay in expressive language as assessed by the Boston Naming Test had no effect on improving the explanatory power of any of the models.

### Association Between Parent-Reported Number of Friends and Emotion Discrimination Skills

The parents were asked about the number of friends visiting their child every week at home and the number of friends the child visited outside the home every week. According to their responses, of the 50 children in the clinical group, 33 had at least one friend who visited them every week, and of these 33 children, 26 had at least one friend whom they visited every week. The mean number of friends visiting the children every week was 1.0 (*SD* = 1.050), and every week they visited 0.7 friends outside the home, on average (*SD* = 1.789). Only nine children had more than one friend who visited them every week, and eight children had more than one friend whom they saw outside their home every week. Overall, a total of 17 children in the whole clinical group had no friends who visited them at home, and 24 children had no friends whom they visited outside the home. The children were then divided into two groups: those who did not have any friends at all and those who had at least one friend. An alarmingly large number—a total of 16 (32%) children out of 50—had no friends at all whom they met every week either at home or outside the home. Therefore, two variables were constructed: children with no friends and children with at least one friend.

While neither sex nor diagnosis had any effect on the results, two significant links were found between the existence of friends and emotion discrimination skills. First, children with a larger delay in their ability to discriminate emotions from meaningful sentences had the fewest number of friends visiting them (ϱ = − 0.295, *p* = 0.038). Children who had no friends visiting them each week (*n* = 17) showed a mean delay of nine percentage points in their ability to discriminate the emotional tone of voice in meaningful sentences compared with those with at least one friend visiting them every week (*n* = 33). The difference in delay between these two groups was significant (*U* = 184.5, *p* = 0.040, *d* = 0.291). The same pattern was found among children who had no friends to visit outside the home (*n* = 24), as they showed significantly greater delay in discriminating the emotional tone of voice in nonsense words (*U* = 212.5, *p* = 0.049, *d* = 0.278) than those (*n* = 26) who had at least one friend to visit outside the home every week. The difference favouring those with at least one friend was, on average, eight percentage points.

Logistic regression analysis was used to identify the risk factors associated with having no friends. The analysis revealed an odds ratio of 1.06; thus, for every percentage point delay in the task of discriminating the emotional tone of voice in nonsense words increased the risk of having no friends to visit by 1.06 points. Additionally, for every percentage point delay in the FEFA 2 test had a similar effect, as it increased the risk of having no friends to visit by 1.05. However, these differences were not statistically significant.

## Discussion

Three of the four hypotheses proposed in this study were fully supported by the results; however, the fourth hypothesis was only partially supported. According to the main results of the present study, the adults perceived the children with neurodevelopmental disorders to have clear social-emotional and behavioural difficulties in daily life situations—not only children with ASD but also those with ADHD and DLD.

In regard to the first hypothesis, parents and professionals perceived the children with neurodevelopmental disorders to have rather severe emotion recognition difficulties in their daily lives and the difficulties did not significantly differ between the three diagnostic groups. Similar results were observed, in accordance with the second hypothesis, on the adults’ answers to the open-ended questions. Parents and professionals reported on many kinds of social-emotional difficulties (and situations in which they occur in the children’s daily lives), especially difficulties in skills of affective ToM. Although the adults were asked to report on emotion recognition difficulties specifically, they included a broader range of social-emotional difficulties in their reports. This suggests close inter-connections between different modalities of social-emotional skills that are undistinguishable to most people. According to previous research, social-emotional difficulties are common in children with neurodevelopmental difficulties, and in children with ASD they are well established (e.g. Baron-Cohen et al., 2009; Kuusikko et al., 2009). More recently, several studies have found similar difficulties in children with ADHD (e.g. Chronaki et al., 2015; Jusyte et al., 2017; Sjöwall et al., 2013; Staff et al., 2022) and DLD (e.g. Griffiths, et al., 2020; Spackman et al., 2005). However, the diagnostic groups of children with ASD, ADHD and DLD have usually been studied separately or comparing no more than two of these groups when studying social-emotional difficulties.

Additionally, children with neurodevelopmental disorders often have behavioural problems (e.g. Charman et al., 2015; Green et al., 2016) and this was prevalent in the present study too, as nearly all of the children were reported to have considerable behavioural problems according to their SDQ scores. These challenges of the children supported the second hypothesis set in the present study. The reported behavioural problems concorded between parents and professionals in only two subscales out of five, however, previous studies have also indicated only modest parent–teacher agreement in SDQ (R. Goodman et al., 2004). Additionally, Borg et al., (2014) found behavioural problems in a Finnish parent-rated child group to be higher than those in the teacher-rated group. These findings were consistent with the total SDQ scores of this study. Based on the parents’ responses, 90% of the children had clear overall behavioural symptoms, whereas based on the professionals’ responses, it was 76%. These findings suggest that the SDQ is useful for providing more accurate overall information when it is administered to informants in different contexts, as not all symptoms may occur in the same way in every situation (R. Goodman et al., 2000; Johnson et al., 2014).

As for the third hypothesis, compared with their TD age peers, the children with neurodevelopmental disorders showed a slight, but statistically significant, delay in all emotion discrimination skills. This finding aligns with many previous studies on children with ASD, ADHD and DLD (e.g. Boucher et al., 2000; Griffiths et al., 2020; Jusyte et al., 2017; Taylor et al., 2015). Furthermore, with respect to the fourth hypothesis, delays in emotion discrimination skills were found to be significant predictors (without intervening factors) for some behavioural problems in daily life in the regression analysis. A particularly interesting finding was that hyperactivity symptoms were explained by the ability to match facial expressions with emotional tone of voice. The vice versa can also be true; children with ADHD have challenges with focusing for a certain amount of time on specific aspects and, therefore, it may be hard for a child with ADHD to match an expression with the corresponding tone of voice. The regression models’ best coefficients of determination ranged from 18 to 38% for explaining the variation in different SDQ subscales. The explanatory power of the delays found in emotion discrimination skills was therefore moderate. The analysis did not reveal emotion discrimination skills as predictors for peer relationship problems. This is probably because peer relationship problems are the result of multiple social-emotional difficulties and not necessarily a direct consequence of poor emotion discrimination skills alone. Furthermore, expressive language delay did not mediate explanatory power on explaining the behavioural problems of the children in this study. Griffiths et al. (2020) found strong associations between early language competence and later emotion recognition skills among 97 children with DLD. As this study included only 13 participants with DLD, the low number of participants could partially explain the non-significant results. Another explanation may be that more complex language skills underlie the development of emotion discrimination skills and their further effect on behavioural problems, although vocabulary is often considered to reflect overall linguistic skills.

One of the most prominent findings of this study was that almost a third of the children had no friends at all, as reported by their parents. Further, almost half of them did not have friends to visit outside the home every week, which was also an alarmingly high proportion. Having no friends to visit was associated with difficulties in discriminating the emotional tone of voice. Based on this result, it can be speculated that discriminating the tone of voice or interpreting the prosodic information of spoken messages is a skill that is somehow important for interacting with peers in social situations. Accordingly, Neves et al. (2021) showed vocal emotion recognition in speech prosody to be associated with social-emotional adjustment (e.g. whether a child played with other children) in 6- to 8-year-old children (*n* = 141), as assessed by teachers. Previous studies have also confirmed that, for example, poor emotional knowledge influences peer acceptance negatively (Izard et al., 2001; Voltmer & Von Salisch, 2017). Furthermore, having no friends leads to a lack of situations in which a child can develop these skills.

### Measures Used in the Present Study

Of the six emotion discrimination tasks employed here, all but the FEFA 2 test were self-constructed. Nevertheless, they have high face validity, as the task types they represent are typically used for assessing emotion discrimination skills (e.g., Boucher et al., 2000; Izard et al., 2001; Taylor et al., 2015; Waddington et al., 2018). When using the KR-20 method, the reliability of the self-constructed tasks used mainly was moderate, although Cronbach’s alpha showed better results when the variables that were expected to measure the same underlying construct were looked at together. Moreover, the emotion discrimination tasks often significantly correlated with each other. Parker et al. (2013) also found the KR-20 score for their six-item task of facial expressions to be low, at 0.41. This may reflect the fact that the concepts measured were not unidimensional; in addition, and in the present study, the age of the children varied from 6 to 10 years, and even the TD children’s skills were still developing. The reliability of a measure may be compromised for several reasons, including the use of only a few items in the tasks, having items of varying difficulty, ceiling or floor effects, differences in difficulty level for different individuals, a small sample size and the attention problems of participants (Parker et al., 2013; Peters, 2014). In the present study, by using results obtained from 106 TD children as a reference, it was possible to determine typical performance in the tasks at each age level. However, the intensity of the emotions used as stimuli was not controlled for, and the posed emotional expressions in the tasks were likely to have been more intense than those encountered in daily life (Motley & Camden, 1988). On the other hand, the children with neurodevelopmental disorders still frequently failed to discriminate even these more intense emotions.

For this study, the Finnish norms for SDQ (Borg et al., 2014) were used. Possibly due to differences in culture and social expectations of children’s behaviour, the cut-off points on different subscales were 1–2.5 points lower than the normative British cut-off points (R. Goodman, 1997). The participants may, therefore, have scored worse than they would have done in Finnish clinical settings, where the British standards are currently used.

### Clinical Implications

Frequent social-emotional and behavioural difficulties in daily life, as well as small but statistically significant delays in emotion discrimination skills, were found in all the assessments. Moreover, based on the findings of this study, due to social-emotional difficulties, children with neurodevelopmental disorders as young as six to ten years of age had already encountered loneliness in their lives. In particular, the ability to discriminate the emotional tone of voice appears, for its part, to be a contributory factor in forming and maintaining friendships. Additionally, problems with ToM may further increase the difficulties these children face when interacting with peers: the difficulties in daily life that parents and professionals reported indicated that affective ToM was a particular challenge for the children. Previous research has suggested that the loneliness that children encounter may also persist into adulthood (A. Goodman et al., 2015; Hintzen et al., 2010). Children with such neurodevelopmental disorders, therefore, need timely intervention that should also include emotion perception training. Every fifth participant in this study had not received any kind of treatment prior to their recruitment. Finally, further research needs to be conducted on intervention outcomes in larger groups of children.

### Conclusions

In this study, parents and professionals perceived children with neurodevelopmental disorders as demonstrating multiple social-emotional and behavioural difficulties that negatively affected their daily lives. The children also showed delayed emotion discrimination skills in comparison with their TD age peers. Some difficulties in emotion discrimination skills may even predict the quality of children’s peer relationships; in the present study, they were predictive of a lack of friends. Almost a third of the participating children had no friends to meet each week, which may indicate that they lacked certain crucial skills required for forming and maintaining friendships. Moreover, from the findings, it is obvious that there are more factors underlying social-emotional and behavioural difficulties than just challenges in emotion discrimination skills.

### Supplementary Information

Below is the link to the electronic supplementary material.

Supplementary file1 (DOCX 20 kb)

Supplementary file2 (DOCX 16 kb)

Supplementary file3 (DOCX 16 kb)
