# Supplementary material for: Social-Emotional and Behavioural Difficulties in Children with Neurodevelopmental Disorders: Emotion Perception in Daily Life and in a Formal Assessment Context
Source: J Autism Dev Disord. 2022 Oct 3;53(12):4744–58. doi: 10.1007/s10803-022-05768-9 (PMC10627915; doi:10.1007/s10803-022-05768-9)
Supplement: Supplementary file 1 — Supplementary file1 (DOCX 20 kb) [file 10803_2022_5768_MOESM1_ESM.docx]

**Online Resource 1**

Article title: Social-emotional and behavioural difficulties in children with neurodevelopmental disorders: Emotion perception in daily life and in a formal assessment context

Journal name: Journal of Autism and Developmental Disorders

Authors: Joanna Löytömäki, Marja-Leena Laakso, Kerttu Huttunen

Corresponding author: Joanna Löytömäki, University of Oulu, Finland, joanna.loytomaki@oulu.fi

*Measures and Their Psychometric Properties in the Present Study as Calculated from the Results of Children with Neurodevelopmental Disorders (n = 50)*

| Measure | Description of measure | Self-constructed or previously validated | Citation | Psychometric properties | Range of total scores |
| --- | --- | --- | --- | --- | --- |
| Data collected  through questionnaires | |  |  |  |  |
| VAS | Parents’ and professionals’ reports about children’s ability to recognise emotions from facial expressions, emotional tones of voice and bodily postures | Self-constructed | Huttunen et al. (2018) | Internal consistency of the parents’ reports: α = 0.779; of the professionals’ reports: α = 0.908 | 0–100 |
| Open-ended questions | Requested descriptions on 1) the situations in which the child was noticed to have emotion recognition difficulties, and 2) how these problems affected the child’s daily life | Self-constructed | Huttunen et al. (2018) | n/a | n/a |
| SDQ | Brief screening questionnaire of behavioural problems and prosocial skills | Previously validated | Goodman, R. (1997) | Internal consistency of the parents’ reports: α = 0.769; and of the professionals’ reports: α = 0.806 | 0–40 |
| Direct  testing |  |  |  |  |  |
| Boston naming test | Expressive vocabulary test | Previously validated | Kaplan et al. (1983); Laine et al. (1997) | Not reported | 0–60 |
| Nonsense words | Discrimination of emotional tone of voice in nonsense words and short sentences | Self-constructed | Huttunen et al. (2018); Löytömäki et al. (2020) | KR-20 = 0.521; correlation with meaningful sentences: *r* = 0.334; internal consistency when together with meaningful sentences: α = 0.498 | 0–18 |
| Meaningful sentences | Discrimination of emotional tone of voice in sentences conveying the same semantic meaning | Self-constructed | Huttunen et al. (2018); Löytömäki et al. (2020) | KR-20 = 0.538; correlation with nonsense words *r* = 0.334; internal consistency when together with nonsense words: α = 0.498 | 0–11 |
| FEFA 2 test | Discrimination of facial expressions from photographs | Previously validated | Bölte et al. (2013) | α = 0.95 in adults Bölte & Poustka (2003) | 0–50 |
| Photo-graphs | Discrimination of facial expressions from photographs | Self-constructed | Huttunen et al. (2018); Löytömäki et al. (2020) | KR-20 = 0.364; correlation with video clips: *r* = 0.669 and with FEFA 2 test: *r* = 0.634; criterion validity when photographs and FEFA 2 test together: α = 0.764 | 0–8 |
| Video clips | Discrimination of facial expressions from dynamic video clips | Self-constructed | Huttunen et al. (2018); Löytömäki et al. (2020) | KR-20 = 0.394; correlation with photographs: *r* = 0.669 and with FEFA 2 test *r* = 0.590; criterion validity when video clips and FEFA 2 test together: α = 0.734 | 0–8 |
| Matching | Matching emotional tone of voice with the correct facial expression | Self-constructed | Huttunen et al. (2018); Löytömäki et al. (2020) | KR-20 = 0.569 | 0–11 |

*Note*. α = Cronbach’s alpha; *r* = Pearson’s correlation, KR-20 = Kuder-Richardson Formula 20, n/a = not applicable
