# Supplementary material for: Social-Emotional and Behavioural Difficulties in Children with Neurodevelopmental Disorders: Emotion Perception in Daily Life and in a Formal Assessment Context
Source: J Autism Dev Disord. 2022 Oct 3;53(12):4744–58. doi: 10.1007/s10803-022-05768-9 (PMC10627915; doi:10.1007/s10803-022-05768-9)
Supplement: Supplementary file 2 — Supplementary file2 (DOCX 16 kb) [file 10803_2022_5768_MOESM2_ESM.docx]

**Online resource 2**

Article title: Social-emotional and behavioural difficulties in children with neurodevelopmental disorders: Emotion perception in daily life and in a formal assessment context

Journal name: Journal of Autism and Developmental Disorders

Authors: Joanna Löytömäki, Marja-Leena Laakso, Kerttu Huttunen

Corresponding author: Joanna Löytömäki, University of Oulu, Finland, joanna.loytomaki@oulu.fi

*Distribution of the Parents’ and Professionals’ Perceptions of Situations in which the Emotion Recognition Difficulties of the Children with Neurodevelopmental Disorders Emerge (with Examples)*

| Question 1: “Describe a situation in which you have noticed difficulties in emotion recognition.” | | | | |
| --- | --- | --- | --- | --- |
| Parents | | | Professionals | |
| Major theme | Number of mentions (%) | Examples | Number of mentions (%) | Examples |
| Affective ToM difficulties | 26 (59%) | “Thinks someone is angry when they are confused.” | 19 (50%) | “Sometimes the child may fail to interpret other people’s facial expressions and tone of voice.” |
|  |  | “The child does not show empathy. For example, if the child’s sister is crying, the child may start laughing.” |  | “Does not pay attention to other people’s facial expressions or react to them.” |
| Cognitive ToM difficulties | 13 (30%) | “The child says that it is impossible to interpret other people’s minds.”  “Does not recognise whether people are laughing with or at him/her.” | 10 (26%) | “Does not understand when the other person wants to stop playing, for example.”  “Does not recognise whether someone is serious or joking.” |
| Social difficulties | 2 (5%) | “Fights easily with siblings when playing.”  “When playing with other children.” | 5 (13%) | “The child has difficulties when playing with others and gets easily left out.”  “Difficulties in interpreting social situations at school” |
| Other problems | 3 (7%) | “The child is afraid of scary computer games and is therefore afraid of passing game stores.” | 4 (11%) | “The child is calm. Does not show confusion.” |

*Note.* ToM = Theory of Mind
