# Supplementary material for: Social-Emotional and Behavioural Difficulties in Children with Neurodevelopmental Disorders: Emotion Perception in Daily Life and in a Formal Assessment Context
Source: J Autism Dev Disord. 2022 Oct 3;53(12):4744–58. doi: 10.1007/s10803-022-05768-9 (PMC10627915; doi:10.1007/s10803-022-05768-9)
Supplement: Supplementary file 3 — Supplementary file3 (DOCX 16 kb) [file 10803_2022_5768_MOESM3_ESM.docx]

**Online resource 3**

Article title: Social-emotional and behavioural difficulties in children with neurodevelopmental disorders: Emotion perception in daily life and in a formal assessment context

Journal name: Journal of Autism and Developmental Disorders

Authors: Joanna Löytömäki, Marja-Leena Laakso, Kerttu Huttunen

Corresponding author: Joanna Löytömäki, University of Oulu, Finland, joanna.loytomaki@oulu.fi

*Distribution of the Parents’ and the Professionals’ Perceptions of What Kind of Daily Life Problems Emotion Recognition Difficulties of Children with Neurodevelopmental Disorders Cause (with Examples)*

| Question 2: “What kind of problems do these emotion recognition difficulties cause the child in daily life?” | | | | |
| --- | --- | --- | --- | --- |
| Parents | | | Professionals | |
| Major theme | Number of mentions | Examples | Number of mentions | Examples |
| Affective ToM difficulties | 7 (15%) | “Other people’s emotions are less important than the child’s own.” | 12 (26%) | “Does not recognise other people’s emotions – does not react.” |
|  |  | “Gets frustrated when they don’t understand if an adult is sad, angry, or surprised.” |  | “In peer relationships, it is hard for the child to understand how others are feeling.” |
| Cognitive ToM difficulties | 11 (23%) | “Finds it difficult to interpret whether the behaviour of friends is intentional. Often interprets behaviour of others as negative.” | 8 (17%) | “Has trouble placing themselves in the place of others.” |
|  |  | “If parents are talking at the same time, the child interprets it as an argument.” |  |  |
| Social difficulties | 19 (40%) | “Does not know how to play with other children.”  “The child has no friends.” | 16 (33%) | “Peer relationships suffer.”  “The child easily gets into trouble in social situations.” |
| Poor emotion regulation | 8 (17%) | “The child often gets angry, especially with their brother.” “Gets in fights easily, does not know how to control their emotions.” | 6 (13%) | “Gets upset and angry easily.”  “Finally, when others are already angry with the child, the child may start (physically) pushing others.” |
| Other problems | 2 (4%) | “The child is unable to verbally express different emotional states (child’s own or others’).” | 4 (9%) | “The child does not know how to tell an adult when they have been punched by someone.” |

*Notes*. ToM = Theory of Mind
